# Genetic liability in individuals at ultra-high risk of psychosis: A comparison study of 9 psychiatric traits

**DOI:** 10.1371/journal.pone.0243104

**Published:** 2020-12-02

**Authors:** Keane Lim, Max Lam, Hailiang Huang, Jianjun Liu, Jimmy Lee

**Affiliations:** 1 Research Division, Institute of Mental Health, Singapore, Singapore; 2 Feinstein Institute of Medical Research, The Zucker Hillside Hospital, New York, New York, United States of America; 3 Stanley Center for Psychiatric Research, The Broad Institute of MIT and Harvard, Cambridge, Massachusetts, United States of America; 4 Genome Institute of Singapore, Singapore, Singapore; 5 Analytic and Translational Genetics Unit, Massachusetts General Hospital, Boston, Massachusetts, United States of America; 6 Yong Loo Lin School of Medicine, National University of Singapore, Singapore, Singapore; 7 Department of Psychosis, Institute of Mental Health, Singapore, Singapore; 8 Neuroscience and Mental Health, Lee Kong Chian School of Medicine, Nanyang Technological University, Singapore, Singapore; Department of Psychiatry and Neuropsychology, Maastricht University Medical Center, NETHERLANDS

## Abstract

Individuals at ultra-high risk (UHR) of psychosis are characterised by the emergence of attenuated psychotic symptoms and deterioration in functioning. In view of the high non-psychotic comorbidity and low rates of transition to psychosis, the specificity of the UHR status has been called into question. This study aims to (i) investigate if the UHR construct is associated with the genetic liability of schizophrenia or other psychiatric conditions; (ii) examine the ability of polygenic risk scores (PRS) to discriminate healthy controls from UHR, remission and conversion status. PRS was calculated for 210 youths (n_UHR_ = 102, n_Control_ = 108) recruited as part of the Longitudinal Youth at Risk Study (LYRIKS) using nine psychiatric traits derived from twelve large-scale psychiatric genome-wide association studies as discovery datasets. PRS was also examined to discriminate UHR-Healthy control status, and healthy controls from UHR remission and conversion status. Result indicated that schizophrenia PRS appears to best index the genetic liability of UHR, while trend level associations were observed for depression and cross-disorder PRS. Schizophrenia PRS discriminated healthy controls from UHR (R^2^ = 7.9%, *p* = 2.59 x 10^−3^, OR = 1.82), healthy controls from non-remitters (R^2^ = 8.1%, *p* = 4.90 x 10^−4^, OR = 1.90), and converters (R^2^ = 7.6%, *p* = 1.61 x 10^−3^, OR = 1.82), with modest predictive ability. A trend gradient increase in schizophrenia PRS was observed across categories. The association between schizophrenia PRS and UHR status supports the hypothesis that the schizophrenia polygenic liability indexes the risk for developing psychosis.

## Introduction

The clinical prodromal syndrome of psychosis, variably termed as ultra-high risk (UHR) [1], clinical high risk [2], or at-risk mental states [3,4] was conceptualised nearly two decades ago to identify and intervene in individuals at risk of developing psychosis, with the expectation of preventing, or at least ameliorating, or delaying the full psychotic manifestation and its devastating consequences [4–10]. This criteria applies to help-seeking young individuals presented with either one of three presentations: (i) attenuated psychotic symptoms, (ii) brief or self-limiting full-blown psychotic symptoms, or (iii) significant decrease in functioning in the context of genetic risk for schizophrenia [11–13].

However, the overall specificity of the UHR construct remains unclear, potentially poses challenges in risk stratification and interventions. While the majority of the conversion being schizophrenia spectrum disorder [14], the rates for transition to psychosis in UHR individuals remains modest, ranging from 18% at 6 months to 32% by 3 years [15]. Moreover, a high percentage of UHR individuals reported non-psychotic comorbidity like depression and anxiety [16]. In light of this, the specificity of the UHR construct has been questioned as a risk for psychosis only or risk for developing any psychiatric condition, with some arguing for a reconceptualization of the UHR construct to extend from a psychosis dimension to a general psychopathology model [17–20].

While accumulating evidence have investigated the UHR construct using neurocognition [21–23], neurobiological substrates [24–26] and environmental risk predictors [27], genetic studies in UHR received relatively lesser attention and were mostly limited to candidate genes [28–32].

Recent surge in new data from genome-wide association studies (GWAS) have made leaps in refining our understanding of the complex genetic architecture of psychiatric traits. This was motivated by the potential of genomics to determine the genetic liability of complex traits, unravel the biological mechanisms that could in turn translate into therapeutics, and to identify clinically high-risk individuals before disease manifestation [33,34]. A direct application of GWAS is the construction of polygenic risk scores (PRS), which sums up risk variants identified from GWAS into a continuous genetic liability score [35–38]. The DNA remains constant throughout the life course, making the PRS an alluring target for pre-emptive clinical treatment [39]; considering that early intervention is associated with better prognosis in psychosis [40,41]. It is hoped that PRS could identify individuals at the extremes of the PRS risk distribution [35,42,43] and complement risk enrichment in tandem with clinical and environmental risk factors [44,45].

Previous studies have reported that the schizophrenia PRS identified and discriminated healthy individuals from individuals with first episode psychosis, schizophrenia and other psychoses [46–50]. More recently, Perkins et al (2020) [51] reported that the schizophrenia PRS discriminated psychosis converters from non-converters in an at-risk sample of European ancestry, while He et al (2019) [52] found no association between schizophrenia PRS and conversion to psychosis. Given that the predictive accuracy of PRS is affected by the ancestry of the training and target dataset, systematic evaluation of PRS in diverse samples is needed to enable equitable application of PRS [53–55].

Therefore, the aim of the present study is (i) to clarify the specificity of the UHR construct by investigating if it more closely approximates the genetic architecture of schizophrenia as initially conceptualised, or if UHR represents a general psychopathology vulnerability as indexed by other psychiatric traits; (ii) to evaluate if PRS could discriminate healthy controls from UHR; (iii) to examine the predictive ability of PRS on UHR remission and conversion status.

## Methods

### Participants

The participants were recruited as part of the Longitudinal Youth at Risk Study (LYRIKS) in Singapore. Details of the study have been reported previously [8,16,56,57]. In brief, LYRIKS is a prospective, observational study on youths, aged 14–29 years, at ultra-high risk of developing psychosis. Recruitment adopted a hybrid approach where both help-seeking and non-help-seeking individuals from the community were approached. Participants were recruited through community social service agencies, educational institutes and mental health services from the Institute of Mental Health in Singapore [58].

Participants were ascertained to be UHR via the Comprehensive Assessment of At-Risk Mental States (CAARMS) [59], with at least one of the three criteria met: (1) vulnerability trait for psychotic illness (having a first-degree relative with psychosis or schizotypal personality disorder), (2) attenuated psychotic symptoms (having subthreshold psychotic symptoms in the past year), and (3) brief limited intermittent psychotic symptoms (had a brief psychotic episode that remitted within a week without the use of any antipsychotic medications in the past year, and showed a deterioration in functioning for at least one month in the past year). The exclusion criteria were: (i) current or past history of psychosis or mental retardation, (ii) current use of illicit substances, (iii) taking mood stabilizers, or (iv) had medical causes associated with their symptoms. Psychiatric history was evaluated with the Structured Clinical Interview for DSM-IV Axis I Disorders [60]. Healthy controls were those who did not fulfil the UHR criteria, had no psychiatric disorder, and had no family history of psychosis.

Participants were followed-up at 6-month intervals for up to 24 months, or until conversion to psychosis. Conversion to psychosis was determined by the fulfilment of CAARMS psychotic threshold criteria of at least one full psychotic symptom lasting more than one week and occurring at least three times a week. Remission status was defined as individuals who met UHR criteria at baseline but no longer fulfil UHR criteria at 24 months. Individuals who met UHR criteria at 24 months (i.e., persistent non-remitters) or had converted to psychosis were categorised as non-remitters. Therefore, individuals at UHR of psychosis were categorised as remitters or non-remitters and converters or non-converters.

Ethics approval for this study was provided by the National Healthcare Group’s Domain Specific Review Board. Written informed consent was obtained from all participants and consent from a legally acceptable representative was obtained for minors below the age of 21 years as required by the local regulations.

### Genotyping, quality control and imputation

The LYRIKS cohort was genotyped on the Illumina Infinium OmniZhongHua-8 BeadChip. Standard quality control procedures (See S1 File for details) were performed in PLINK 1.9 [61] to exclude single nucleotide polymorphisms (SNPs) with minor allele frequency (MAF) < 0.01, call rate < 0.98, Hardy-Weinberg equilibrium p-value < 1e-06; and exclude individuals with mismatch between recorded and genotyped sex, and related individuals. Cryptic relatedness was identified with identity by descent method (pi-hat > 0.2).

As the LYRIKS cohort consisted of individuals of Han Chinese descent, Malay descent and South Indian descent, population ancestries were verified by conducting principal component analysis against the Singapore Genomic Variation Project (SGVP) [62] and the 1000 Genomes phase 3 reference panel [63]. Samples with more than four standard deviations away from the SGVP reference panel, along the first ten ancestral principal components were excluded (S1 File; S1 and S2 Figs in S2 File).

Imputation was conducted separately for each ancestry with Minimac3 (MaCH) [64] against the full 1000 genomes phase 3 reference panel [65]. The imputed SNPs underwent the second round of quality control with the same parameters above, and filtered for imputation quality score > 0.9. The imputed data was merged on shared SNPs for full sample analysis, resulting in a total of 3,349,959 high quality SNPs.

### Polygenic risk scores

Schizophrenia polygenic risk scores (PRS) was calculated for each individual as the weighted sum of risk alleles using GWAS summary statistics from the CLOZUK and Psychiatric Genomics Consortium schizophrenia wave-2 meta-analysis (CLOZUK-PGC2) [66] and the PGC East-Asian schizophrenia meta-analysis (PGC SCZ-EAS) [53] as discovery samples.

PRS was also calculated using GWAS summary statistics of eight psychiatric traits: major depressive disorder (MDD) [67–69], bipolar disorder [70], anxiety disorder [71], obsessive-compulsive disorder [72], anorexia nervosa [73], autism spectrum disorder [74], attention deficit hyperactivity disorder [75] and cross-disorder [76]. For MDD, PRS was calculated using three discovery datasets, denoted here as MDD-2013 [67], MDD-Converge [68], and MDD-2019 [69], to examine its predictive ability based on the different ancestry and MDD phenotypic definition employed in these GWAS studies. The MDD-2013 and MDD-Converge ascertained MDD based on clinically derived DSM diagnostic criteria, while the MDD-2019 utilised a mix of self-reported and full diagnostic criteria. The MDD-2013 and MDD-2019 were conducted in samples of European ancestry, while the MDD-Converge was conducted in East-Asian samples.

To select the most informative and independent markers, the following parameters were performed for each summary statistic: p-value informed clumping of r^2^ > 0.1 with 500kb windows; EUR and EAS individuals from the 1000 genomes phase 3 were extracted as LD reference panel for clumping variants in the respective summary statistics; SNPs in the major histocompatibility complex (MHC; chr6:25–35Mb) were removed due to the complex linkage disequilibrium pattern in this region; and only SNPs with imputation quality score > 0.9 were selected.

PRS were calculated for ten p-value thresholds (P_T_ ≤ 5 x 10^−08^, 1 x 10^−05^, 1 x 10^−04^, 1 x 10^−03^, 0.01, 0.05, 0.1, 0.2, 0.5, 1). PRS were standardised using means and standard deviations from healthy controls. The PRS were constructed using PRSice-2 [77,78].

Power calculation for PRS was implemented in R package ‘AVENGEME’ [79,80], and the genetic covariance and proportion of variance explained by genetic effects were estimated with the package. Prevalence was assumed to be 0.01 [81,82].

### Statistical analysis

To investigate the genetic liability of the UHR construct, PRS calculated using summary statistics derived from GWAS of schizophrenia and eight other psychiatric traits were performed using logistic regression with the first ten ancestral principal components, age and gender as covariates on UHR-Healthy control status. The proportion of variance explained by PRS was calculated with Nagelkerke’s pseudo-R^2^, by comparing the full model (i.e., PRS with covariates) with the reference model (i.e., covariates only). A Bonferroni correction of *p* = 0.0042 (0.05/12 GWAS discovery dataset) was applied.

Ordinal logistic regression was performed to examine if PRS was associated with remission (Healthy controls x Remitters x Non-remitters) or conversion to psychosis (Healthy controls x Non-converters x Converters), adjusted for the first ten ancestral principal components, age and gender.

The discriminatory power of the PRS was investigated with the area under the receiver operator curve (AUC), using the best fit p-value threshold model, and compared with a reference model (i.e., covariates only) and a full model (i.e., PRS and covariates). To aid the visualisation of PRS distribution, the sample was divided into quintiles by PRS, and estimated for UHR-Healthy control odds ratio (OR) and 95% confidence interval. This analysis was also adjusted for the first ten ancestral principal components, age and gender.

These statistical analyses were performed on IBM SPSS version 23 [83] and figures were plotted in R v3.4.3 packages [84–86].

## Results

### Sample characteristics

The study sample characteristics are presented in Table 1. A total of 210 individuals (n_UHR_ = 102, n_Control_ = 108) were available for analysis after quality control procedures (See S1 File for details). Within UHR, 49 (48%) were remitters and 53 (52%) were non-remitters; 90 (88.2%) were non-converters and 12 (11.8%) were converters.

**Table 1:** Sample characteristics across groups.

	Healthy Controls	UHR	Remitters	Non-remitters	Persistent Non-remitters	Non-Converters	Converters
N	108	102	49	53	41	90	12
Age, years	22.07 (3.48)	21.84 (3.61)	21.65 (3.49)	22.02 (3.75)	22.22 (3.88)	21.91 (3.66)	21.33 (3.31)
Gender (M/F)	69/39	71/31	32/17	39/14	29/12	61/29	10/2
Ethnicity							
Chinese	74 (68.5)	77 (75.5)	34 (69.4)	43 (81.1)	35 (85.4)	69 (76.7)	8 (66.7)
Malay	22 (20.4)	19 (18.6)	12 (24.5)	7 (13.2)	3 (7.3)	15 (16.7)	4 (33.3)
Indian	12 (11.1)	6 (5.9)	3 (6.1)	3 (5.7)	3 (7.3)	6 (6.7)	0 (0)

*Note*. UHR = Ultra high risk to psychosis; M/F = Males/Females Values in cells represent mean (SD) or n (%), unless otherwise stated.

### Genetic liability of UHR with schizophrenia PRS and other psychiatric traits

**Fig 1:**
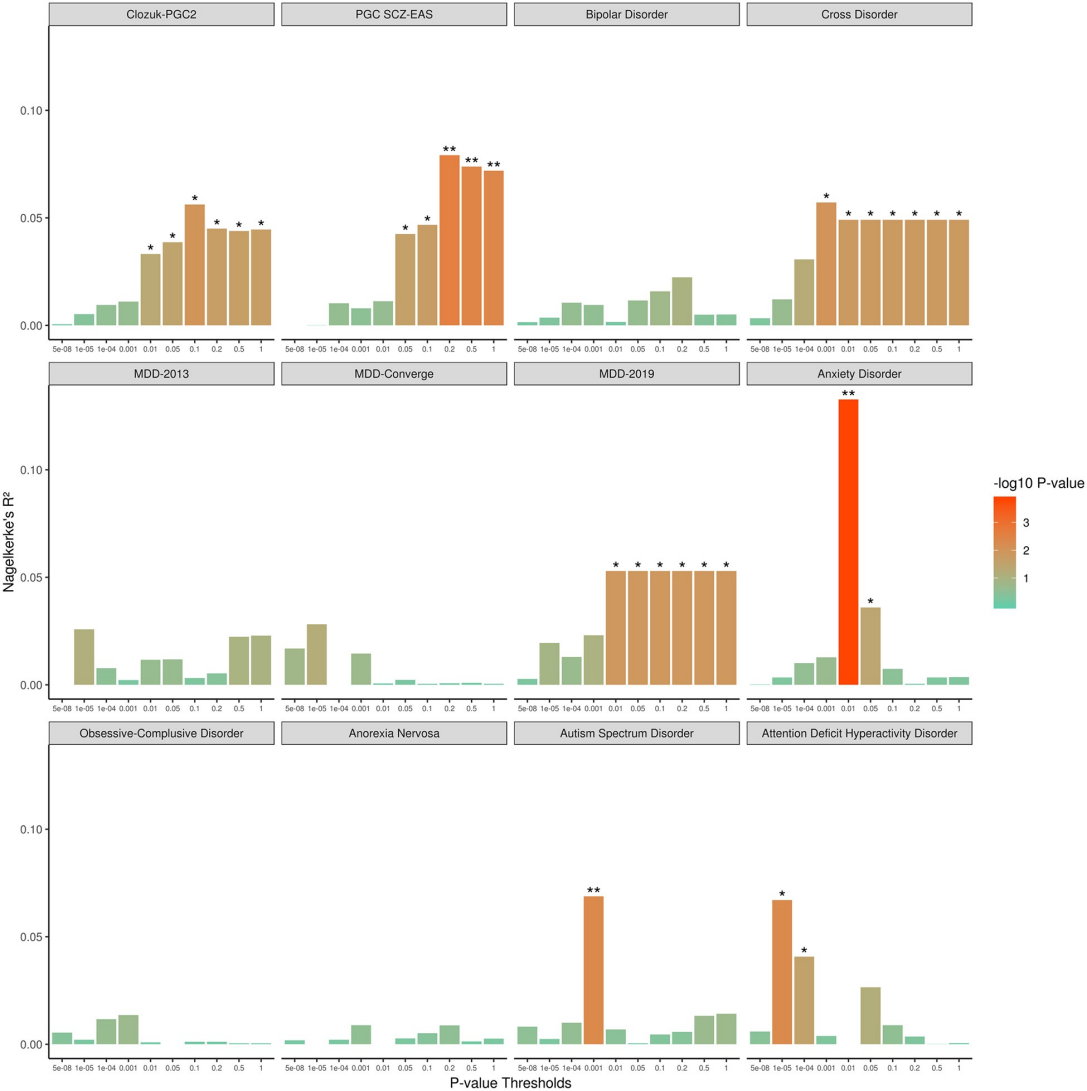
Polygenic prediction of psychiatric traits with UHR-Healthy control status in Han Chinese. Note. *Unadjusted p-value < 0.05. **Bonferroni correct p-value significance.

The genetic liability of the UHR status examined using the Han Chinese group, the largest ethnic group in this cohort, revealed that the schizophrenia PRS best discriminated UHR-Healthy control status (Fig 1). Amongst the other psychiatric traits, only the MDD-2019 and cross-disorder showed trend level associations, with increasing variance explained as the number of SNPs increased across the p-value thresholds (Fig 1). As the PGC SCZ-EAS PRS showed better discrimination in our Asian sample, compared to the CLOZUK-PGC2 PRS (Fig 1; S1–S3 Tables in S3 File), subsequent analyses described herein used the PGC SCZ-EAS PRS.

**Fig 2:**
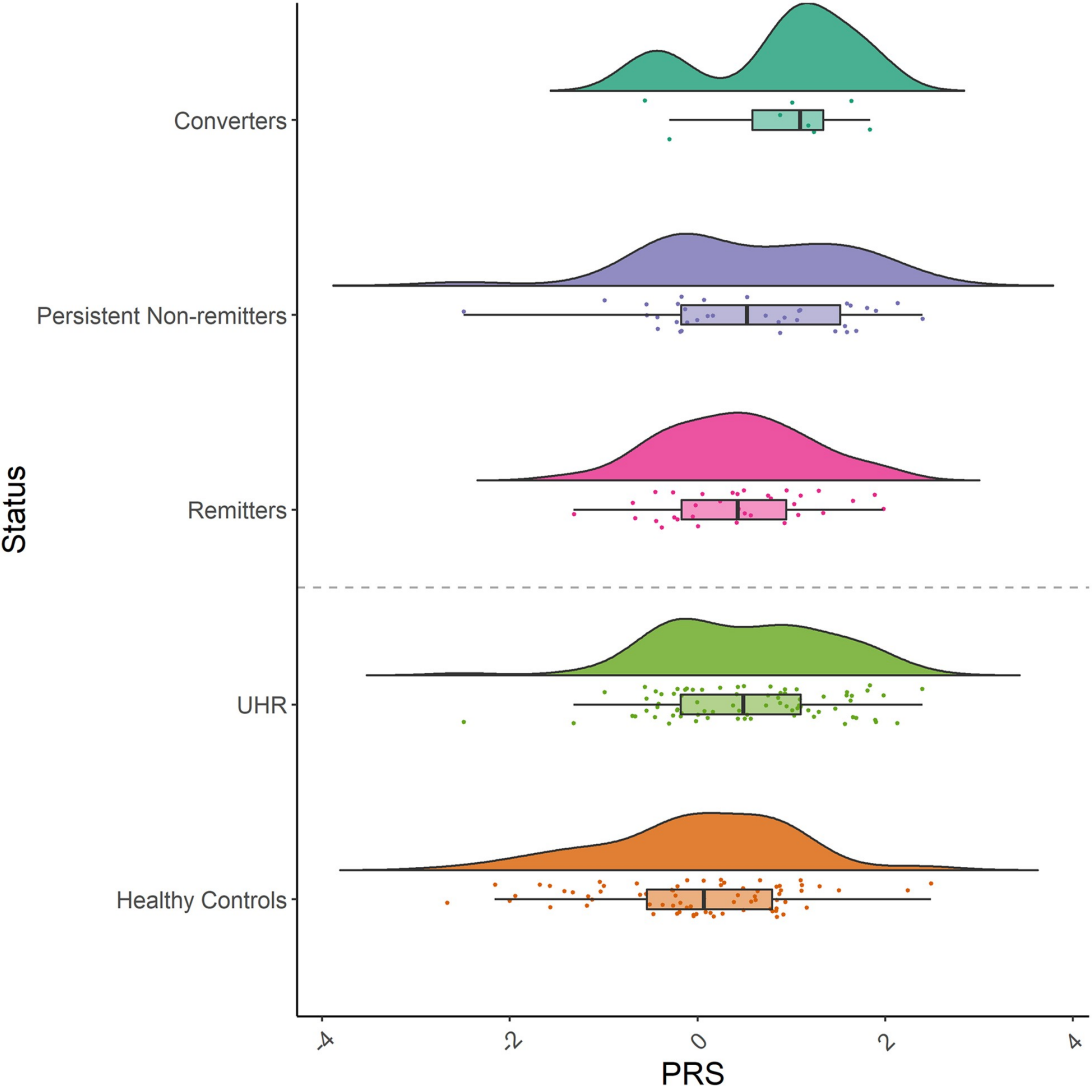
Raincloud plot of PGC SCZ-EAS standardized polygenic risk scores (PRS) in the Han Chinese group. Raincloud plots are presented for healthy controls, UHR, remission and conversion status. The raincloud plot aids data visualisation by combining a split-half violin plot, raw jittered data points, and central tendency of median through a boxplot.

The association of the PGC SCZ-EAS PRS and UHR-Healthy control status was further examined using the total sample and stratified sample. Significant differences in PGC SCZ-EAS PRS were observed between UHR-Healthy control status (*p* = 0.001) (Fig 2). In the total sample, the PGC SCZ-EAS PRS discriminated UHR-Healthy control status (Nagelkerke’s R^2^ = 5.1%, *p* = 4.17 x 10^−3^, *p*_T_ = 1, OR = 1.96, 95% CI = 1.24–3.11) (S1 and S2 Tables in S3 File). Further UHR-Healthy control analysis based on stratification by the two major ethnic group within the cohort revealed that the PGC SCZ-EAS PRS was predictive of UHR-Healthy control status in individuals of Han Chinese descent (Nagelkerke’s R^2^ = 7.9%, *p* = 2.59 x 10^−3^, *p*_T_ = 0.2, OR = 1.82, 95% CI = 1.23–2.69, power = 0.79; Fig 1), but was not associated with UHR-Healthy control status in individuals of Malay ancestry, after accounting for Bonferroni correction of *p* = 0.008 (0.05/6 leave-one-out iterations) (S1 and S2 Tables in S3 File). Additional leave-one-out analysis indicated that the PGC SCZ-EAS PRS showed better predictive ability in individuals of Han Chinese descent compared to other ethnicities in this cohort (S1 and S2 Tables in S3 File).

To rule out the possibility that the UHR-Healthy control association observed was driven by the subgroup of converters which have the highest mean PRS, a sensitivity analysis of UHR-Healthy control was performed by removing this subgroup of converters from the UHR sample. Results indicated that PRS still discriminated UHR-Healthy control status without the converters subgroup, with a similar pattern of association (S4 Table in S3 File).

### Schizophrenia PRS associations with remission status

**Fig 3:**
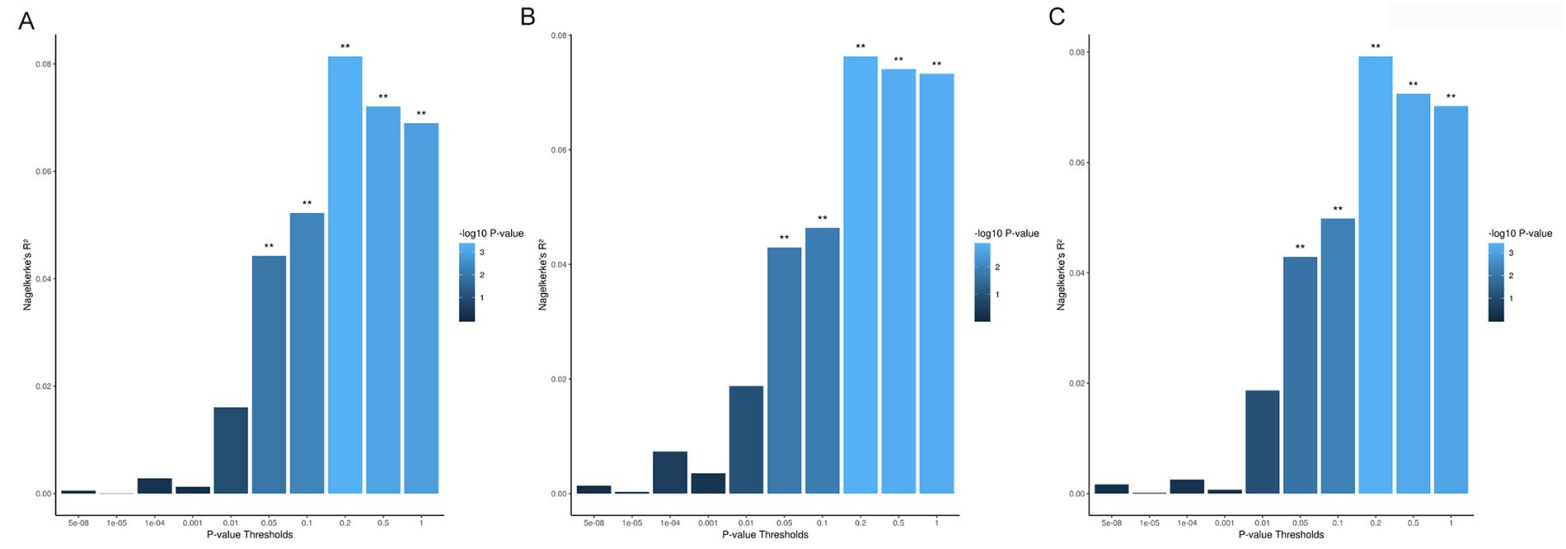
Proportion of variance explained by PGC SCZ-EAS polygenic risk scores (PRS) in the Han Chinese. (A) Healthy controls vs remission status. (B) Healthy controls vs conversion status. (C) Healthy controls vs remitters vs persistent non-remitters vs converters. Note. **P-value significance < 0.05.

PRS discriminated healthy controls from remission status (Nagelkerke R^2^ = 8.1%, *p* = 4.90 x 10^−4^, *p*_T_ = 0.2, OR = 1.90, 95% CI = 0.28–1.00) (Fig 3A; S5 Table in S3 File). PRS was higher in non-remitters, followed by remitters, then controls (Fig 2; S3 Fig in S2 File). Post-hoc analysis indicated significant difference in PRS between non-remitters and controls (*p* = 0.004), but not between remitters and controls (*p* = 0.09), and within UHR (*p* > 0.05).

### Schizophrenia PRS associations with conversion status

PRS discriminated healthy controls from conversion status (Nagelkerke R^2^ = 7.6%, *p* = 1.61 x 10^−3^, *p*_T_ = 0.2, OR = 1.82, 95% CI = 0.23–0.98) (Fig 3B; S6 Table in S3 File). PRS was higher in converters, followed by non-converters, then controls (Fig 2; S3 Fig in S2 File). Post-hoc analysis indicated significant difference in PRS between converters and controls (*p* = 0.04), and non-converters and controls (*p* = 0.01). No difference was observed within UHR (*p* > 0.05).

### Schizophrenia PRS associations with remission and conversion status

To further evaluate the associations found between healthy controls with remission and conversion status, an additional secondary analysis was performed. The non-remitters group was re-categorised as persistent non-remitters (i.e., individuals who still met UHR criteria at 24 months, but did not convert to psychosis) and converters. Ordinal logistic regression (Healthy controls x Remitters x Persistent non-remitters x Converters) revealed PRS discriminated these categories (Nagelkerke R^2^ = 7.9%, *p* = 4.57 x 10^−4^, *p*_T_ = 0.2, OR = 1.89, 95% CI = 0.28–0.99) (Fig 3C; S7 Table in S3 File). PRS was higher in converters, followed by persistent non-remitters, remitters, then healthy controls (Fig 2).

### Predictive ability of schizophrenia PRS

**Fig 4:**
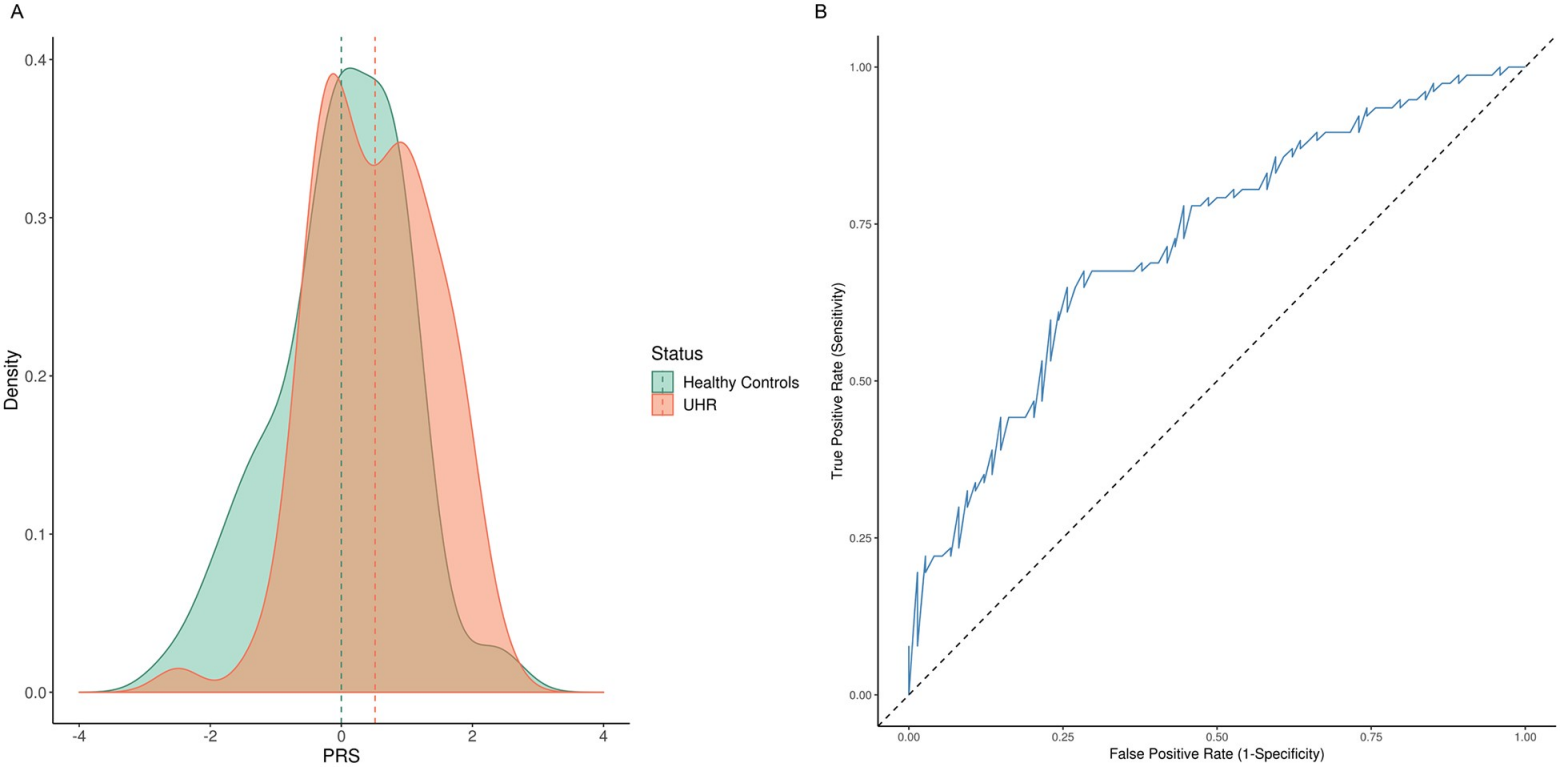
Discriminatory ability of polygenic risk scores (PRS) in Han Chinese case-controls individuals. (A) Density distribution plot. (B) Area under curve (AUC) plot. Vertical dotted lines in panel (A) present mean PRS for each status.

AUC analysis on the discriminatory ability of PRS on UHR-Healthy control status was 0.67 in the reference model. The AUC increased to 0.72 when PRS was added to the model (Fig 4). In a series of AUC analyses, the discriminatory ability of PRS was also compared between healthy controls with remitters (AUC = 0.69), persistent non-remitters (AUC = 0.78) and converters (AUC = 0.93) in the full model.

To investigate the effect of PRS on UHR status, the sample was divided into quintiles based on PRS profile and UHR-Healthy control OR was calculated with the middle quintile as reference. A trend increase in risk of UHR status was observed with greater PRS (S4 Fig in S2 File; S8 Table in S3 File). Compared to the middle quintile, individuals in the fifth quintile had the highest OR (OR = 2.4, 95% CI = 0.71–8.44), while individuals in the first quintile had the lowest OR (OR = 0.48, 95% CI = 0.12–1.82). Generally, the UHR-Healthy control ratio and percentage of non-remitters and converters increased across PRS quintiles (S5 Fig in S2 File; S9 Table in S3 File).

## Discussion

This study utilised the PRS to examine the genetic liability of UHR and its predictive ability. Our results showed that the East-Asian schizophrenia PRS, amongst other psychiatric traits, appears to best approximate the genetic liability of UHR, and discriminated healthy controls from UHR individuals, remission status and conversion status in our cohort.

Polygenic prediction with eight other psychiatric traits [67–75] did not discriminate UHR-Healthy control status, following multiple corrections. Amongst these psychiatric traits, the MDD-2019 [69] and cross-disorder [76] were most predictive of UHR-Healthy control status, showing comparable variance explained to that of CLOZUK-PGC2 [66]. However, this was not observed in the MDD-2013 [67] and MDD-Converge [68] PRS. A plausible explanation for the differences in MDD predictive ability could be attributed to phenotypic definition of MDD employed in the GWAS analysis, where MDD-2019 included a broad phenotyping definition of MDD, while MDD-2013 and MDD-Converge employed a strict phenotyping definition based on full diagnostic criterion of MDD. Moreover, it has been recently suggested that the broad phenotyping definition of MDD indexes a general vulnerability to poor mental health, rather than specific to MDD alone [87,88]. Given the high prevalence of comorbidity and manifestation of non-psychotic symptoms in the UHR population [16], it is speculated that the trend UHR-Healthy control status discriminated by MDD-2019 could be driven by a vulnerability to general psychopathology in the UHR group.

Nevertheless, the results of the current study appear to suggest that the UHR construct could be indexed by liability for schizophrenia, while further clarification is needed on how MDD or general psychopathology is related to the UHR construct. This, however, is caveated by the differing sample size and ancestry in the discovery dataset, requiring further replication. Moreover, the association between the genetic liability for these psychiatric traits and the variation in manifestation of psychotic and non-psychotic symptoms, its onset and trajectory remains to be explored in the UHR population.

The PRS explained 7.9% of the variance in our Han Chinese UHR-Healthy control analysis. This is comparable with previous studies on European ancestry which reported that schizophrenia PRS explained 9.4% variance in first episode psychosis [47] and 9% in psychotic disorders [46], and 9.4% variance on the liability scale in a UHR sample [51]. Further stratification of UHR for remission and conversion status revealed that PRS was associated with UHR status and its subgroups when compared to healthy controls. Within UHR, while there were no significant differences in PRS between remitters and non-remitters, and converters and non-converters, genetic heterogeneity between these categories is suggestive. Particularly, across categories, a gradient increase in mean PRS was observed from controls, to remitters, to converters. This graded pattern of PRS across categories is intuitive, plausibly reflecting the degree of schizophrenia genetic liability with risk of developing psychosis. This PRS dosage effect is congruent with that reported previously in a UHR European sample [51]. Alternatively, the lack of significant separation in PRS within UHR categories may be moderated by environmental risk factors or endophenotypes that may not otherwise be captured by genetic loci derived from a case-control GWAS [89]. Further evaluation of the risk or protective factors underpinning remission or conversion status through interactions between PRS and environmental factors, and how it exerts on the expression of endophenotypes remains to be elucidated [27,90]. Additionally, the interaction between gene and environment, not otherwise captured by a case-control GWAS loci, such as methylation changes [91,92] or blood-based gene expression signatures [93,94] may complement UHR and conversion to psychosis predictability.

Consistent with the literature on the discriminative accuracy of PRS in psychiatry [46,49], a moderate AUC of 0.72 was observed in the current study. This is in contrast to potential clinical utility of PRS reported for common diseases [95]. While the current predictive power precludes the use of PRS for clinical practice in psychiatry, findings of the current study showed promise on the potential utility of PRS in identifying strata of individuals at greater risk of developing psychosis. Specifically, a higher percentage of remitters and converters were reported at the higher extreme of the PRS distribution. This subgroup of individuals could benefit from prioritising treatment plans. Moreover, as the sample size of discovery dataset and yet to be identified rare and common variants increase, the refinement of the PRS is expected [35,79]. PRS prediction models could also be optimised with the inclusion of environmental variables related to the phenotype of interest [96].

Differences in PRS discriminative ability was evident across discovery samples. Consistent with a previous study [53], the PRS performance in our Asian cohort was better with the PGC SCZ-EAS than the CLOZUK-PGC2. Moreover, while limited by the small sample size of each ethnicity in our cohort, PRS models mainly explained variance in individuals of Han Chinese descent but was poor for other ethnic groups, albeit larger sample size of the Han Chinese descent relative to the other ethnic groups. Future studies with larger samples of other ethnic groups are warranted to further examine the predictability of PRS in these ethnic groups. Despite the larger discovery sample size of the CLOZUK-PGC2 compared to the PGC SCZ-EAS, differences in PRS predictive ability could be attributed to differences in allele frequency distributions and LD structures across ancestry [53]. This finding further highlights the importance of greater ancestry diversity in genetic studies, so as to optimise the envisioned potential of PRS [55].

### Strengths and limitations

This cohort of UHR is unique in that individuals were antipsychotic naïve and free of illicit substance use [8], which may otherwise mar the association between status and PRS. The investigation of UHR individuals here extends previous knowledge on how schizophrenia PRS indexes the lability for psychosis across the psychosis spectrum, from prodromal phase as evaluated in the current study, to eventual diagnosis of schizophrenia or psychosis investigated previously [46,47,49]. Nevertheless, further research on the generalisability of these findings to other ethnicities is warranted as our cohort is relatively small and composed of Asian individuals.

However, it should be noted that the conversion rate is low. In the Han Chinese subgroup, only 8 (10.4%) UHR individuals converted to psychosis. This may have limited the variation in severity which could contribute to the lack of clear PRS delineation within UHR. Moreover, while this cohort was followed-up longitudinally for 24 months, a longer prospective follow-up could further refine the UHR remission and conversion status, particularly if subgroups of non-remitter converted to psychosis, or if remitters met criteria for UHR again.

Additionally, the number of variants in the genotyping array could influence the PRS predictive power. Given that the array used in this study provides better coverage for the Han Chinese group compared to the Malay or Indian ethnic groups, genotyping array with wider coverage for the Malay and Indian ethnic groups is needed to examine the generalisability of PRS prediction in these groups.

The results of this study should also be interpreted with the following caveats. First, apart from PGC SCZ-EAS and MDD-Converge, all other discovery datasets were of European ancestry. Therefore, no definitive conclusion may be drawn from the lack of associations between our Asian UHR sample and some other psychiatric traits. Second, the differing sample size in the discovery and target datasets may influence the discriminative power to detect PRS associations with the current UHR sample. Future studies with diverse ancestry discovery datasets and larger target datasets are warranted to examine and replicate these findings.

## Conclusions

This study supported the hypothesis that the UHR status appears to more closely approximate schizophrenia risk than other psychiatric traits. The schizophrenia PRS also discriminated healthy controls from UHR status, showing a trend gradient of schizophrenia polygenic liability across remission and conversion status. Further research on stratification of these individuals could potentially facilitate genomics as a tool in the precision medicine toolbox for psychiatry. However, our study highlighted a need to study PRS in diverse ancestries to enable equitable application of PRS in identifying UHR individuals.

## Supporting information

S1 File (DOCX)

S2 File (DOCX)

S3 File (XLSX)
