# Supplementary material for: Genetic liability in individuals at ultra-high risk of psychosis: A comparison study of 9 psychiatric traits
Source: PLoS One. 2020 Dec 2;15(12):e0243104. doi: 10.1371/journal.pone.0243104 (PMC7710117; doi:10.1371/journal.pone.0243104)
Supplement: S1 File — (DOCX) [file pone.0243104.s001.docx]

**Genetic Liability in Individuals at Ultra-High Risk (UHR) of Psychosis: A Comparison Study of 9 Psychiatric Traits**

**Genotyping**

Venous whole blood was collected from participants of the Longitudinal Youth at Risk Study (LYRIKS) cohort into EDTA tubes. Genotyping procedures were carried out by the Genome Institute of Singapore. Genomic DNA was extracted and genotyped on the Illumina Infinium OmniZhongHua-8 BeadChip kit with the Infinium HD super assay.

**Genome-wide Quality Control**

Prior to quality control procedures, genotype information was available for 242 individuals (N_UHR_ = 119, N_Control_= 123), with 878,291 single nucleotide polymorphism (SNPs). Genome-wide quality control procedures were carried out using PLINK 1.9 [[1]](http://[1]) (www.cog-genomics.org/plink/1.9/). SNPs were excluded if call rate < 0.98; Hardy-Weinberg equilibrium p-value < 1 x 10^-6^; minor allele frequency < 0.01. Sample were checked mismatch between recorded and genotyped sex. Sample were also excluded if call rate < 0.98; inbreeding coefficient > 0.2. SNPs position and alleles were aligned to the Genome Reference Consortium Human Build 37 (GRCh37) [[2]](https://paperpile.com/c/GUyTPk/YlGYk) and the 1000 genomes reference panel phase 3 [[3]](https://paperpile.com/c/GUyTPk/LihMK). SNPs also were removed if there was allele mismatch, invariant SNPs, and ambiguous ‘A-T’ and ‘C-G’ allele.

Cryptic relatedness was examined with identity by state/identity by descent plots to exclude related samples (i.e., first and second-degree relatives). Pi-hat of 0.2 (Z_0_ > 0.625) was used to filter out related samples. Population stratification was determined by first generating a linkage disequilibrium (LD) independent variant set, then subjected to PCA in PLINK 1.9.

Prior to PCA, Chromosome 8 inversion region (chr8: 7000000-15000000) and Major Histocompatibility Complex region (MHC; chr6: 25000000-35000000) were removed. LD pruning was then conducted with a threshold of 100 SNPs 50 SNPs window and r^2^ = 0.2. The pruned dataset was merged with the Singapore genomic variation project (SGVP) [[4]](https://paperpile.com/c/GUyTPk/ZFUvw), and 20 principal components were then generated via PLINK 1.9. The LYRIKS cohort ancestries (Han Chinese, Malay or Indian) was ascertained by mapping against the SGVP (Supplementary Figs. 1-2). Samples with more than four standard deviations away from the SGVP reference panel, along the first ten ancestral principal components were excluded.

After quality control, 210 samples remained with 534,654 SNPs. Phasing and imputation were performed for each ancestry group separately. Phasing was performed with ShapeIt [[5]](https://paperpile.com/c/GUyTPk/kbuTN) for individuals of Han Chinese and Indian ancestry against the 1000 genomes phase 3 East-Asian and South Asian ancestry reference panel respectively. As the Malay ancestry was not in the 1000 genomes reference panel, these samples were phased using the full 1000 genomes panel. Imputation was performed using Minimac3 (MaCH) [[6]](https://paperpile.com/c/GUyTPk/0qrQt) with the full 1000 genomes phase 3 reference panel. The imputed SNPs underwent the second round of quality control with the same parameters above and filtered for imputation quality score > 0.9. The imputed data was merged on shared SNPs for full sample analysis and only biallelic SNPs were retained, resulting in a total of 3,349,959 high quality SNPs.

**References**

1. [Chang CC, Chow CC, Tellier LC, Vattikuti S, Purcell SM, Lee JJ. Second-generation PLINK: rising to the challenge of larger and richer datasets. Gigascience. 2015;4: 7.](http://paperpile.com/b/GUyTPk/jT4vv)

2. [Church DM, Schneider VA, Graves T, Auger K, Cunningham F, Bouk N, et al. Modernizing reference genome assemblies. PLoS Biol. 2011;9: e1001091.](http://paperpile.com/b/GUyTPk/YlGYk)

3. [1000 Genomes Project Consortium, Auton A, Brooks LD, Durbin RM, Garrison EP, Kang HM, et al. A global reference for human genetic variation. Nature. 2015;526: 68–74.](http://paperpile.com/b/GUyTPk/LihMK)

4. [Teo Y-Y, Sim X, Ong RTH, Tan AKS, Chen J, Tantoso E, et al. Singapore Genome Variation Project: a haplotype map of three Southeast Asian populations. Genome Res. 2009;19: 2154–2162.](http://paperpile.com/b/GUyTPk/ZFUvw)

5. [O’Connell J, Gurdasani D, Delaneau O, Pirastu N, Ulivi S, Cocca M, et al. A general approach for haplotype phasing across the full spectrum of relatedness. PLoS Genet. 2014;10: e1004234.](http://paperpile.com/b/GUyTPk/kbuTN)

6. [Das S, Forer L, Schönherr S, Sidore C, Locke AE, Kwong A, et al. Next-generation genotype imputation service and methods. Nat Genet. 2016;48: 1284–1287.](http://paperpile.com/b/GUyTPk/0qrQt)
