# Supplementary material for: Genetic liability in individuals at ultra-high risk of psychosis: A comparison study of 9 psychiatric traits
Source: PLoS One. 2020 Dec 2;15(12):e0243104. doi: 10.1371/journal.pone.0243104 (PMC7710117; doi:10.1371/journal.pone.0243104)
Supplement: S2 File — (DOCX) [file pone.0243104.s002.docx]

**Genetic Liability in Individuals at Ultra-High Risk (UHR) of Psychosis: A Comparison Study of 9 Psychiatric Traits**


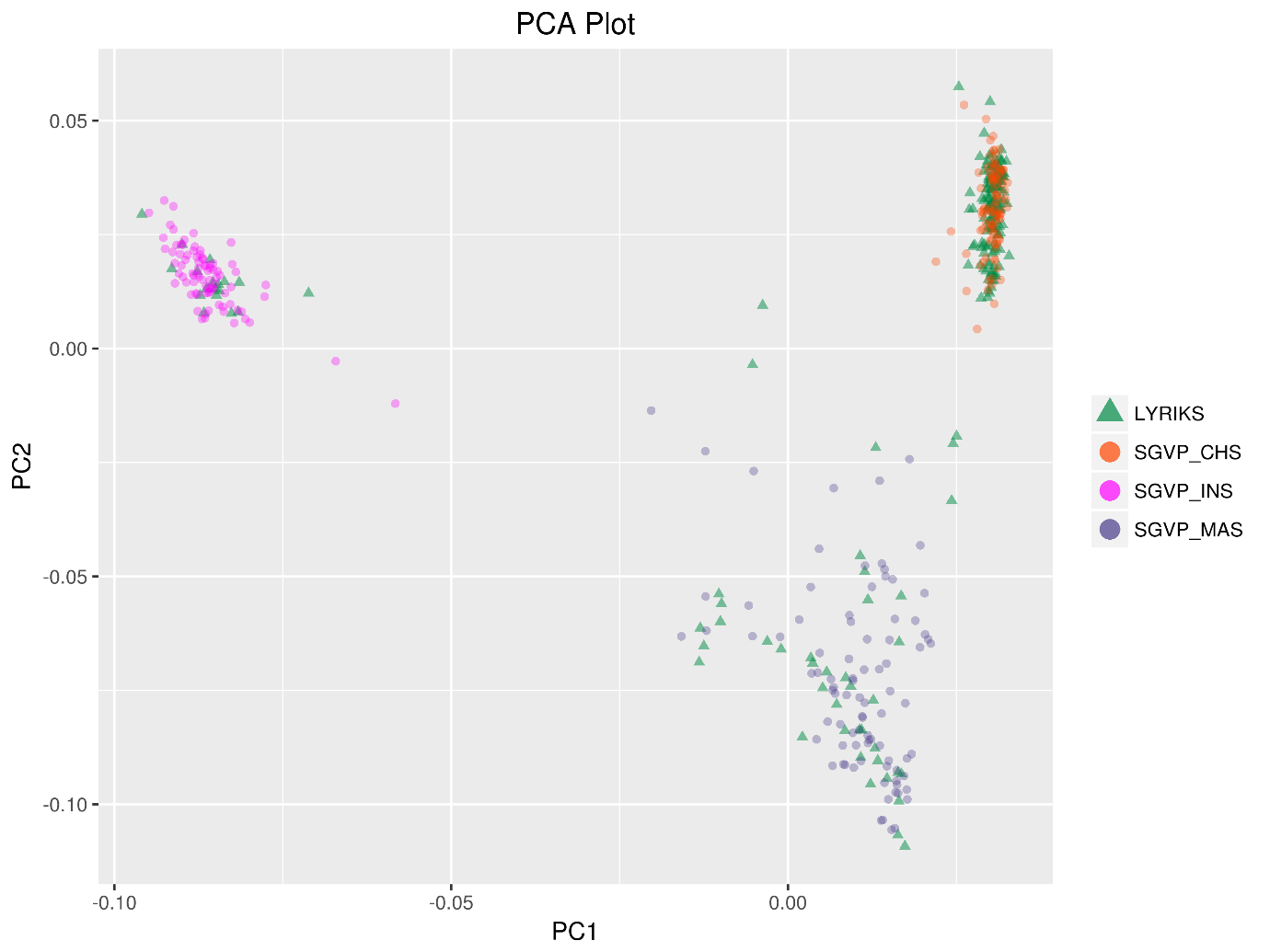


**S1 Fig. PCA ancestry verification of LYRIKS mapped against the Singapore Genomic Variation project reference (SGVP).** SGVP_CHS = Han Chinese, Singapore; SGVP_INS = Indian, Singapore; and SGVP_MAS = Malay, Singapore.


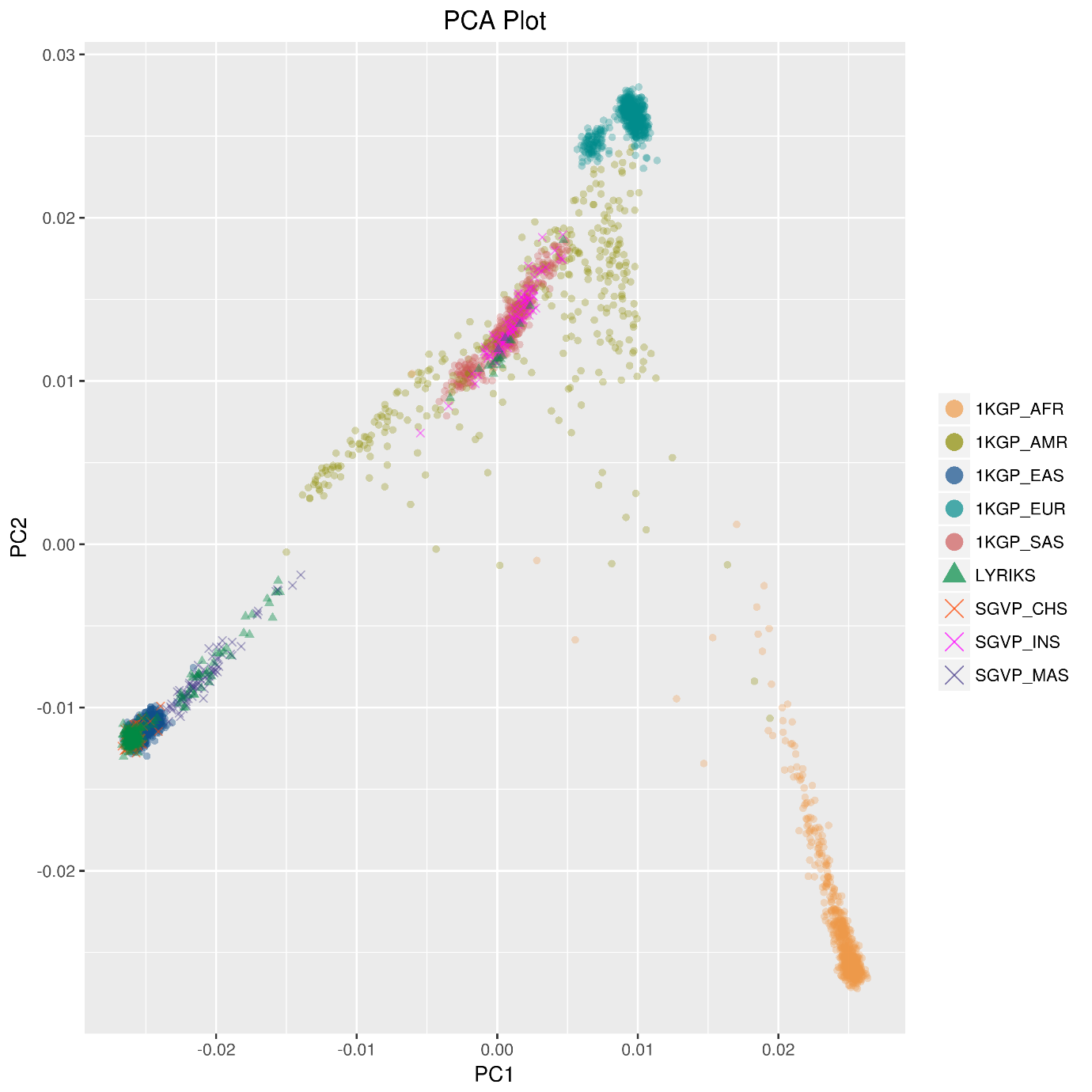


**S2 Fig. PCA plot of LYRIKS mapped against the Singapore Genomic Variation Project (SGVP) and the 1000 genomes phase 3 reference panel (1KGP).** SGVP_CHS = Han Chinese, Singapore; SGVP_INS = Indian, Singapore; and SGVP_MAS = Malay, Singapore.


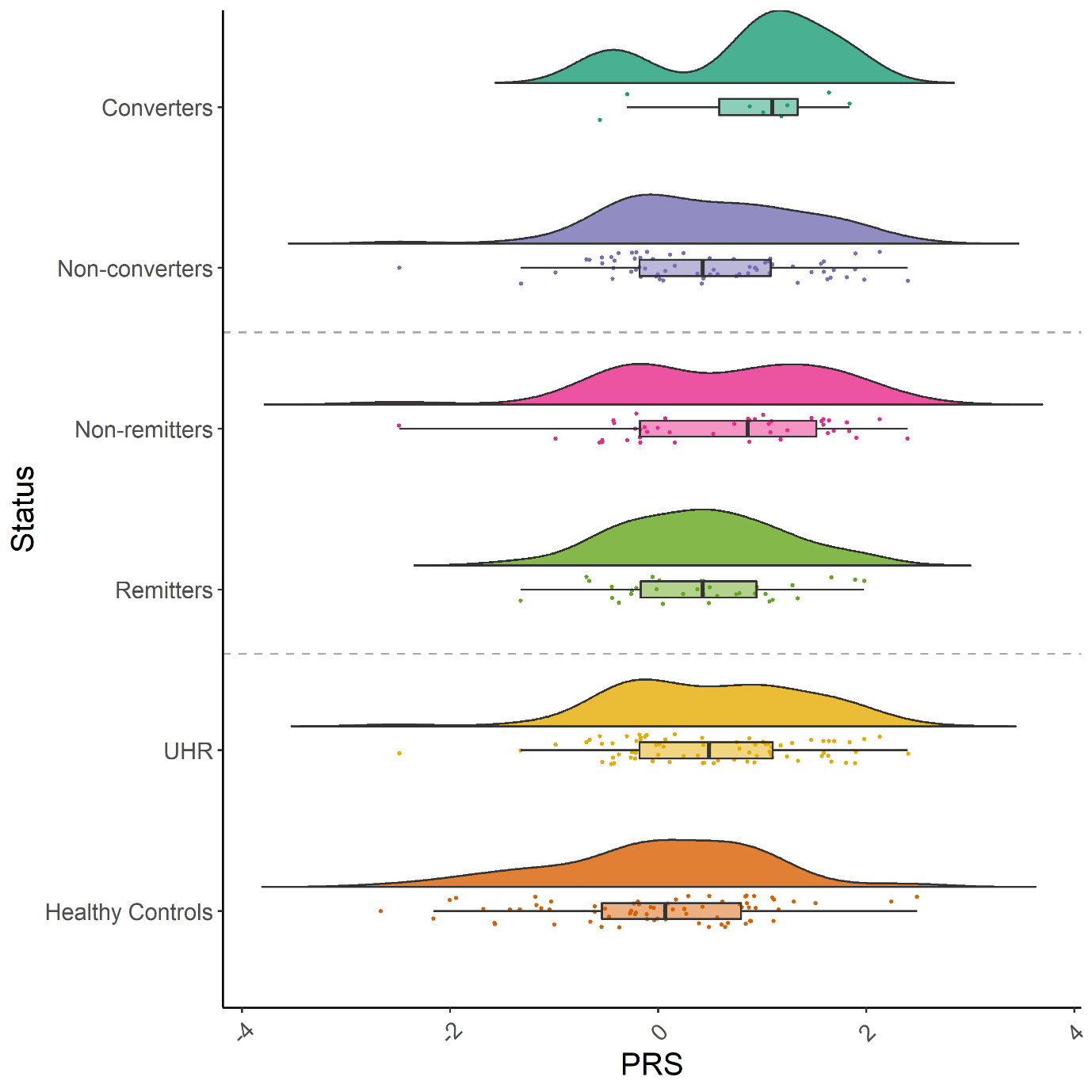


**S3 Fig. Raincloud plot of PGC SCZ-EAS standardized polygenic risk scores (PRS) in the Han Chinese group for healthy controls, UHR, remission and conversion status.** The raincloud plot aids data visualisation by combining a split-half violin plot, raw jittered data points, and central tendency of median through a boxplot.

**
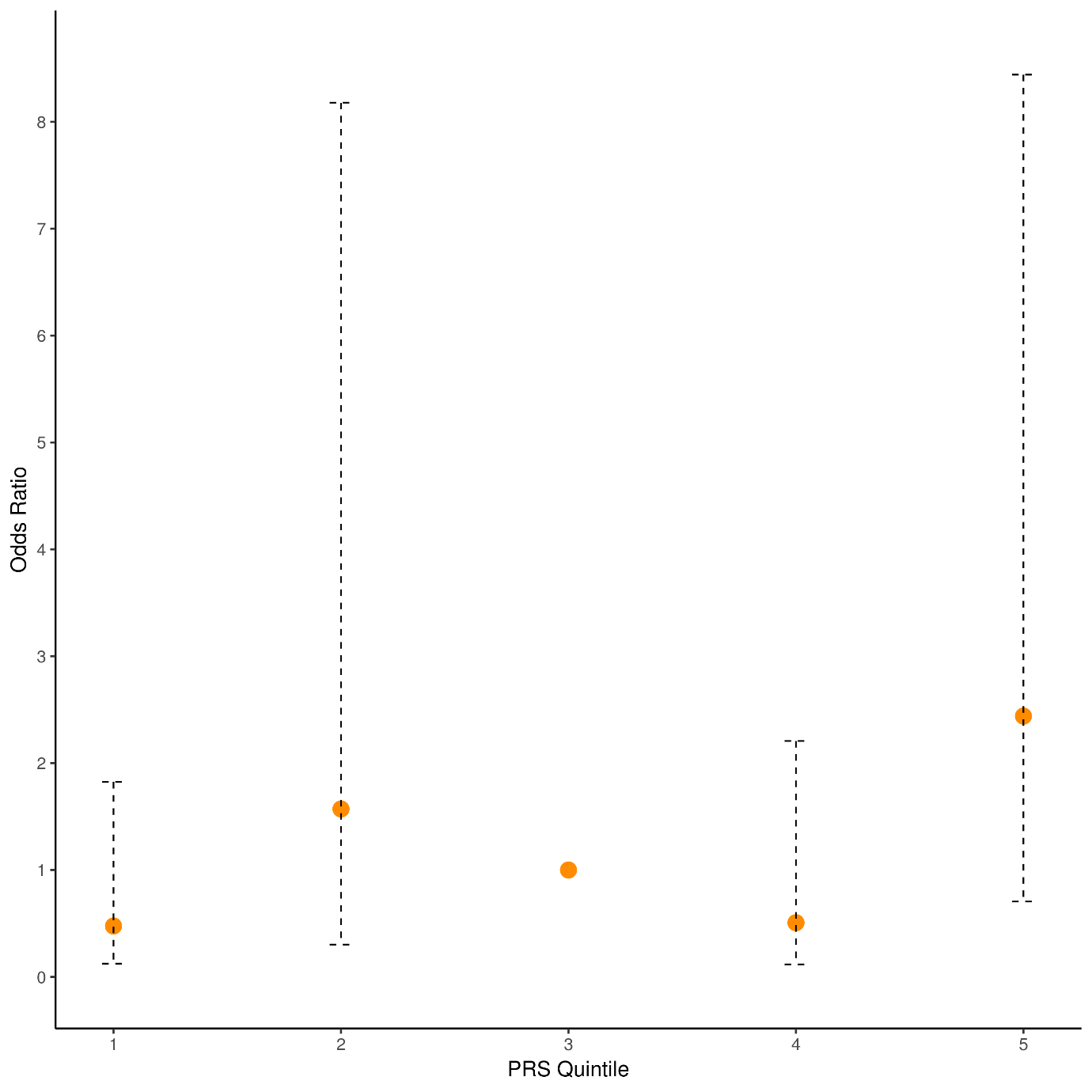
**

**S4 Fig. Odds ratio of case-control status in Han Chinese individuals by PGC SCZ-EAS polygenic risk scores (PRS) quintiles.** Each quintile is compared with the middle (3^rd^) quintile. The vertical dotted lines represent 95% confidence intervals.

**
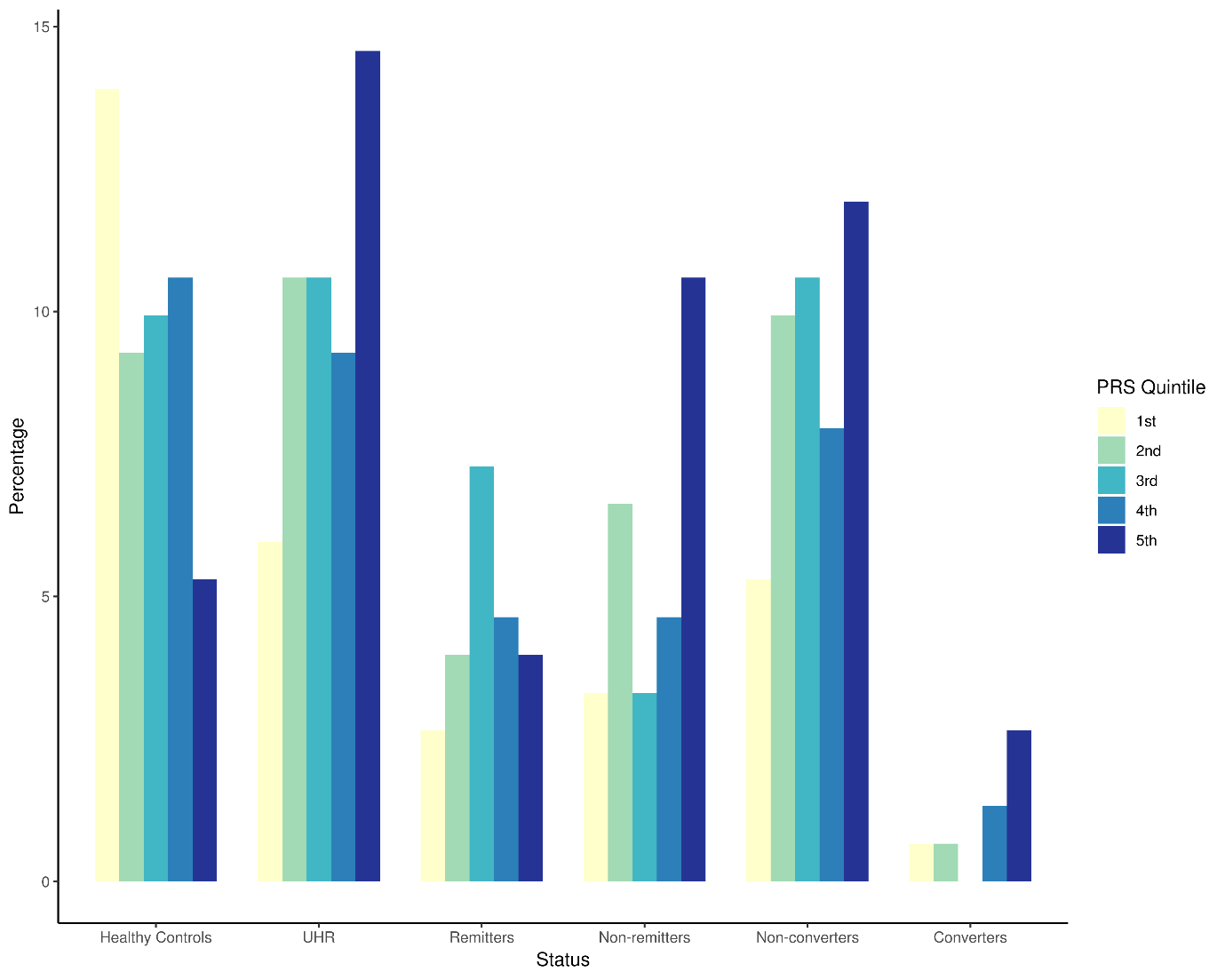
**

**S5 Fig. Percentage distribution of PGC SCZ-EAS polygenic risk scores (PRS) in Han Chinese individuals across quintiles for healthy controls, UHR, remission and conversion status.** The percentage for each group at each quintile is calculated as the proportion of individuals by the total sample.
